# Wrist Pulse Rate Monitor Using Self-Injection-Locked Radar Technology

**DOI:** 10.3390/bios6040054

**Published:** 2016-10-26

**Authors:** Fu-Kang Wang, Mu-Cyun Tang, Sheng-Chao Su, Tzyy-Sheng Horng

**Affiliations:** Department of Electrical Engineering, National Sun Yat-sen University, Kaohsiung 80424, Taiwan; p940281@gmail.com (M.-C.T.); scps312@gmail.com (S.-C.S.); jason@ee.nsysu.edu.tw (T.-S.H.)

**Keywords:** wrist pulse rate monitor, continuous-wave (CW) radar, self-injection-locked (SIL) radar, bistatic radar architecture

## Abstract

To achieve sensitivity, comfort, and durability in vital sign monitoring, this study explores the use of radar technologies in wearable devices. The study first detected the respiratory rates and heart rates of a subject at a one-meter distance using a self-injection-locked (SIL) radar and a conventional continuous-wave (CW) radar to compare the sensitivity versus power consumption between the two radars. Then, a pulse rate monitor was constructed based on a bistatic SIL radar architecture. This monitor uses an active antenna that is composed of a SIL oscillator (SILO) and a patch antenna. When attached to a band worn on the subject’s wrist, the active antenna can monitor the pulse on the subject’s wrist by modulating the SILO with the associated Doppler signal. Subsequently, the SILO’s output signal is received and demodulated by a remote frequency discriminator to obtain the pulse rate information.

## 1. Introduction

Heart rate variability (HRV) refers to the variation in the time interval between heartbeats. Because of HRV’s potential of providing critical information regarding mental and physical treatments and fitness tracking [1,2,3,4], the use of wearables to conduct long-term monitoring of heart/pulse rates of subjects has become increasingly commonplace [5], and various consumer products such as Fitbit wristbands and the Apple Watch have been developed in succession.

Table 1 compares common techniques of vital sign detection. Accelerometers measure the variations in resistance, voltage, or capacitance caused by change in shape or position of an inertial device to obtain the motion of an object. They can detect acceleration, vibration, and angle of tilt. Recently, in combination with various smart handheld devices, the application of accelerometers has been successful in detecting the rise and fall of the chest to obtain respiratory information with low system complexity and a 1.65 mW power consumption [6]; however, the sensitivity of this technology is insufficient for monitoring the heart rate.

When applying pressure to specific solid materials, a voltage difference is created across the opposite sides of the pressured surface. This conversion of mechanical force to electricity is called the “direct piezoelectric effect”. After modifying the piezoelectric material and the thickness of the substrate, a piezoelectric device can be placed at the wrist radial artery of the subject to measure the pulse rate [7]. In electrocardiography (ECG), electrodes are attached near the heart, and the potential differences captured by the electrodes are amplified by an instrumentation amplifier to obtain bioelectrical signals transmitted through nervous conduction. Consisting of a P wave, a QRS complex, and a T wave, the waveforms of such signals can show depolarization and repolarization of the atria and ventricles [8]. Piezoelectric devices and ECG both require direct skin contact to detect displacement and voltage variation. The contact may cause skin irritation and makes similar methods unsuitable for burn patients and patients with external bleeding. Although dry electrodes are expected to replace conventional wet electrodes that use gel, their widespread use is difficult because the high impedance between skin and electrode often causes the instability of signal conduction [9]. Moreover, the difficulty is further intensified by the requirement of an instrumentation amplifier with high gain, low noise, and low power consumption to amplify tiny output signals for piezoelectric and ECG devices.

Another common noninvasive detection technology is photoplethysmography (PPG). First proposed in the 1930s [10], it mainly comprises a light source and sensors, illuminating the skin with red, green, or infrared light. Hemoglobin (Hgb) in the blood affects the skin’s reflection of light, and by detecting the contraction of blood vessels, it is possible to obtain the pulse rate. In recent years, applications of pulse rate monitoring have broadened from fingertip measurement and wrist-worn fitness trackers to non-contact facial detection [11,12,13,14,15]. However, the complexity of system architecture also leads to excessive cost and power consumption. Another drawback of PPG, however, is that it is susceptible to environmental and physical interruption in its detection [16], such as due to the tightness of the wristband, the shape of the wrist, the location of blood vessels, skin color, ambient light, and skin features including moles, birthmarks, and tattoos, all of which can affect the accuracy of detection. Moreover, detection cannot be performed on clothed bodies.

In light of this, the electromagnetic wave is introduced because of its ability to penetrate non-metal materials and its beyond visual range. Radar technology was first used in non-contact vital sign detection in 1970 [17], and its applications later extended to disaster rescue [18] and remote healthcare [19,20]. Based on the signals emitted, radar systems can be categorized into pulse radars and continuous-wave (CW) radars. Pulse radars emit a series of radio frequency (RF) pulse sequences and obtain the distance and motion information of an object according to the time delay and waveform fluctuations of echo signals. With a programmable switch [21], pulse radars can emit and receive radio frequency (RF) signals only at specific intervals, thereby avoiding electromagnetic interference (EMI) issues, reducing clutter in the environment, and decreasing power dissipation. In 2013, Lin proposed a wearable pulse radar device [22], with a pulse duration of 5 ns, to conduct near-field detection on wrist veins. However, because the system required concurrency control and two independent antennas to prevent a radar blind zone in which the pulse rate could not be detected, the design involved high complexity and high cost.

CW radars consistently emit signals and receive signals reflected from objects, and therefore do not suffer from blind zones. However, the clutter problem still needs to be solved. Sources of clutter include additional stationary objects and transmitter-receiver coupling in the system. Common solutions to the clutter problem include introducing a dual-antenna architecture, implementing a single antenna with high-isolation components to prevent crossover issues [23], and generating signals with the same amplitude with clutter but with an inverted phase [24]. These methods pose challenges to the battery life and the device form factor that falls within a wearable size. To the best of my knowledge, no wrist-worn pulse monitoring device based on conventional CW radar technology currently exists.

In 2010, self-injection-locked (SIL) radar was first proposed and used in non-contact detection of vital signs [25]. The SIL radar is essentially a type of CW radar that transmits a signal from an oscillator to the chest of the subject and then receives the reflected signal for injection locking the oscillator. The Doppler effect created by respiration and heartbeats causes the oscillator’s output signals to be frequency-modulated, and vital signs of the subject can be obtained once the output signals are demodulated by the back-end frequency discriminator. In addition to outstanding sensitivity, this architecture has the advantage of not being easily affected by TX-RX coupling, making it suitable for single-antenna operation [26]. Furthermore, this technology allows the SIL oscillator (SILO) and the frequency discriminator, with an antenna installed for each of them, to be placed at different locations in a bistatic architecture [27]. Implementing the features above, a chest-worn health monitor was recently reported to be capable of monitoring the respiration, heart rate, and pace of an exercising subject at the same time by processing demodulated signals using joint time frequency analysis and moving average filtering [28].

In Section 2, this study compares remote vital sign monitoring capabilities of conventional CW radar and SIL radar, and proves that the latter achieves a similar sensitivity with much lower power consumption than the former. Section 3 discloses and analyzes a wrist pulse rate monitor based on a bistatic SIL radar architecture. Experiment results of pulse rate monitoring are presented in Section 4. The monitor implements an SILO-based active antenna on a wristband to modulate the transmitted signal using the Doppler signal generated by pulse motion, and utilizes an injection-locked oscillator (ILO)-based frequency discriminator to receive and demodulate the transmitted signal at a remote location. Section 5 of this study is its conclusion.

## 2. Comparison between Conventional CW Radar and SIL Radar

**Figure 1:**
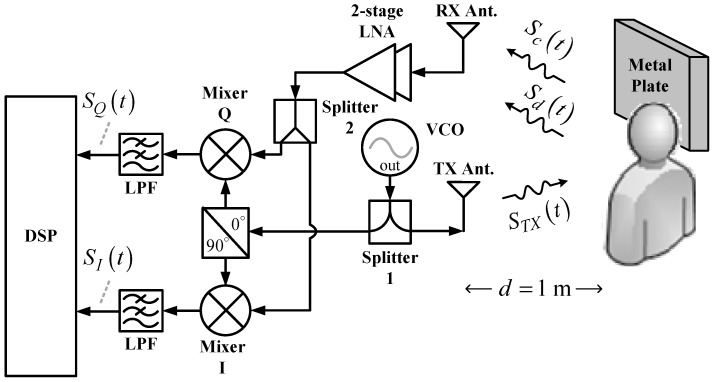
Conventional CW radar for non-contact vital sign monitoring.

As shown in Figure 1, the conventional CW radar architecture comprises one transmit (TX) antenna and one receive (RX) antenna, both of which are panel antennas with an antenna gain of 12 dBi, one two-stage low-noise amplifier (LNA) with a power gain of 23 dB, one voltage-controlled oscillator (VCO), two power splitters, one quadrature mixer, two lowpass filters (LPFs), and one digital signal processor (DSP). The operating principle is as follows. The VCO generates continuous RF signals that are divided into two outputs by Splitter 1. One output of the signal passes through the quadrature power splitter, which further divides the signal into two output signals to pump the quadrature mixers. The other output signal, $S_{TX}\left(t\right)$, from Splitter 1 is radiated by the TX antenna toward the chest of the subject one meter from the radar. Next to the subject, an additional 60 × 60 cm^2^ metal plate is installed for stronger signal reflection. Therefore, the RX antenna will receive two types of signals. One is the Doppler signal, $S_{d}\left(t\right)$, from the chest of the subject. Respiration and heartbeats cause the chest to rise and fall, which phase-modulates the emitted signal $S_{TX}\left(t\right)$. The other is the clutter signal, $S_{c}\left(t\right)$, from other body parts of the subject and the metal plate. Stationary during the detection process, $S_{c}\left(t\right)$ is an attenuated and time-delayed version of $S_{TX}\left(t\right)$. After being received by the RX antenna, $S_{d}\left(t\right)$ and $S_{c}\left(t\right)$ go through a two-stage LNA and are then divided into two outputs by Splitter 2 for feeding the input ports of the quadrature mixer. After the lowpass filter (LPF) filters out the high-order intermodulation components from the output signals of the quadrature mixer, the in-phase signal $S_{I}\left(t\right)$ and quadrature signal $S_{Q}\left(t\right)$ are output to the DSP with a built-in analog-to-digital converter (ADC) for subsequent signal processing and analysis from which the vital sign information of the subject can be obtained.

**Figure 2:**
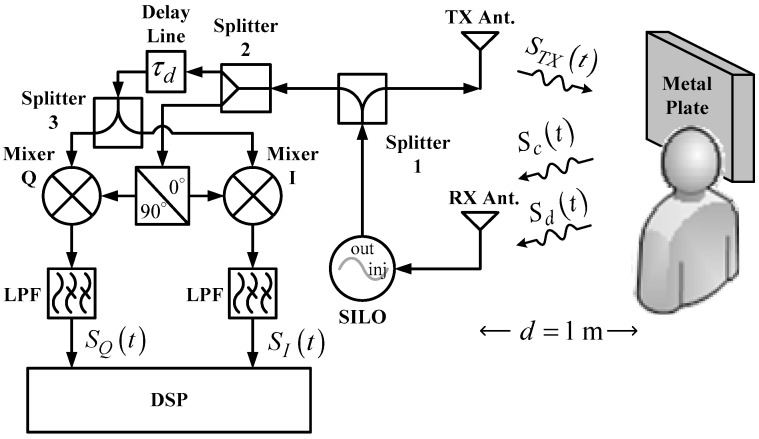
SIL radar for non-contact vital sign monitoring.

Figure 2 shows the system architecture of the SIL radar. As opposed to Figure 1, this circuit adopts a SILO, which has an additional injection port, as compared to conventional VCO, for injection signal input. Moreover, the radar uses an additional power splitter and a delay line that provides a delay time of $\tau_{d}$. This architecture, however, omits two-stage LNA; in other words, the SILO is the only active component in the system. In operation, the reflection signals received by the RX antenna are injected into the SILO. Meanwhile, the rise and fall of the subject’s chest causes the SILO to output frequency modulated (FM) signals as a result of the SIL effect [25]. The other output of Splitter 1 is connected to a quadrature mixer-type frequency discriminator [29]. Splitter 2 divides the FM signals from Splitter 1 into two outputs; one is used as the local oscillator (LO) signals for the quadrature mixer, and the other is entered into the cable assembly used as the delay line. The delay line provided 27 ns of time delay and 2.5 dB of power loss. The delayed signals are further divided by Splitter 3 into two outputs that are fed into the input port of the quadrature mixer. The mixer’s output signals pass through LPFs, which output $S_{I}\left(t\right)$ and $S_{Q}\left(t\right)$ to the DSP.

**Figure 3:**
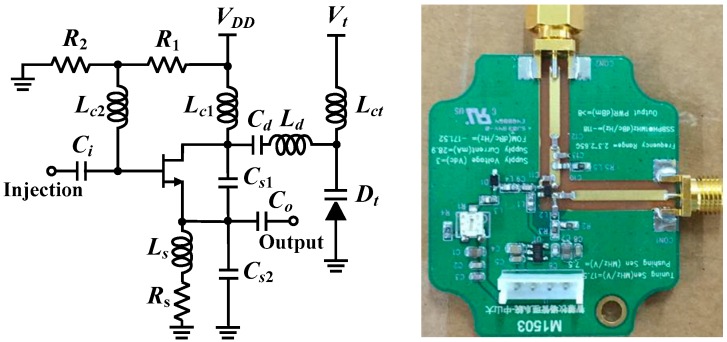
Schematic and photograph of the S-band SILO.

The prototypes based on conventional CW and SIL radar technology operate at the 802.11 b/g/n frequency band of 2.4–2.5 GHz to take advantage of using low-cost Wi-Fi components and antennas. The VCO in Figure 1 and the SILO in Figure 2 are self-made circuits. Figure 3 shows the schematic and photograph of the SILO circuit, which has a tuning range from 2.3 to 2.65 GHz and an output power of 10 dBm. Notably, the design of the SILO uses the Clapp configuration along with an injection port connected to the gate of the transistor. When the injection port is terminated by 50 ohm, it can be used as a regular VCO. The rest of the components in the two radar prototypes are basically the same commercial off-the-shelf items. In Figure 1 and Figure 2, because the SIL radar uses one extra power splitter, the LO powers of the mixers in the two systems are 4 and 1 dBm, respectively. The mixers used in the experiment are ZEM4300+ made by Mini-Circuits, with a conversion loss of about 9 dB.

**Figure 4:**
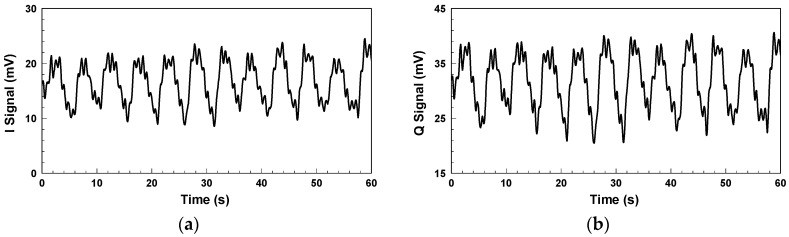
Baseband signals for non-contact vital sign detection using the conventional CW radar: (**a**) I signal; (**b**) Q signal.

Figure 4 shows the in-phase/quadrature (I/Q) baseband signals measured with the architecture in Figure 1. Because respiration causes more significant chest displacement than heartbeats, the waveform in Figure 4 mainly reflects respiration, and the minor fluctuations are the results of heartbeats. Other notable aspects are clutter signals in the environment and imperfections in the components, such as the LO-RF feedthrough of the mixer, mismatch between components, and TX-RX coupling. The additional DC offset caused by TX-RX coupling can be eliminated by the optimization method proposed in [30].

(1) $$Opt\left(V_{DC\_I},V_{DC\_Q},A\right)=\text{min}\left|\sqrt{{\left(S_{I}\left(t\right)-V_{DC\_I}\right)}^{2}+{\left(S_{Q}\left(t\right)-V_{DC\_Q}\right)}^{2}}-A\right|$$

**Figure 5:**
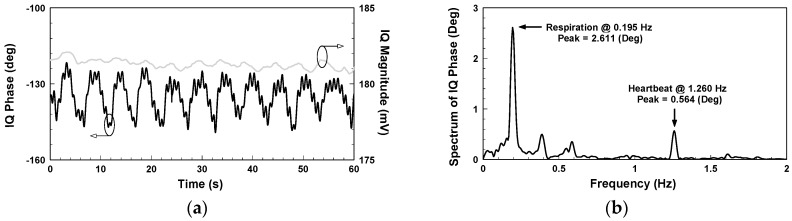
Vital sign detection results using the conventional CW radar: (**a**) IQ magnitude and phase; (**b**) Spectrum of IQ phase in Figure 5a.

In Equation (1), $V_{DC\_I}$ and $V_{DC\_Q}$ are the DC offsets of the IQ baseband signals. Because of path and phase differences, these two DCs are usually not the same. A is the ideal magnitude. Substituting $V_{DC\_I}$ with 159.38 mV and $V_{DC\_Q}$ with −79.77 mV into the following formulas

(2) $$\text{IQ Phase}={\text{tan}}^{-1}\left(\frac{-\left(S_{Q}\left(t\right)-V_{DC\_Q}\right)}{\left(S_{I}\left(t\right)-V_{DC\_I}\right)}\right)$$

(3) $$\text{IQ Magnitude}=\sqrt{{\left(S_{I}\left(t\right)-V_{DC\_I}\right)}^{2}+{\left(S_{Q}\left(t\right)-V_{DC\_Q}\right)}^{2}}$$

yields the demodulated phase and magnitude, represented by the black and grey lines, respectively, in Figure 5a, where the magnitude is around 181 mV. Performing fast Fourier transform on the IQ phase in Figure 5a gives the frequency spectrum shown in Figure 5b, where the respiratory and heart rates are, respectively, 11.7 and 75.6 beats per minute and the peak values 2.611 and 0.564 degrees, respectively.

**Figure 6:**
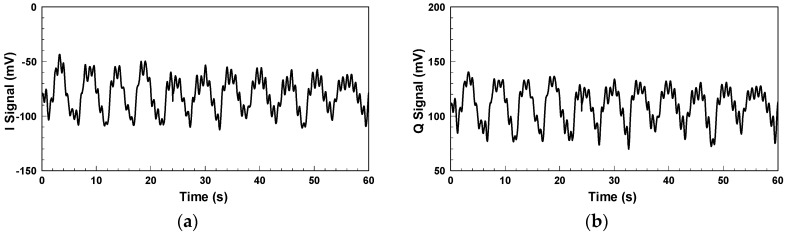
Baseband signals for non-contact vital sign detection using the SIL radar: (**a**) I signal; (**b**) Q signal.

**Figure 7:**
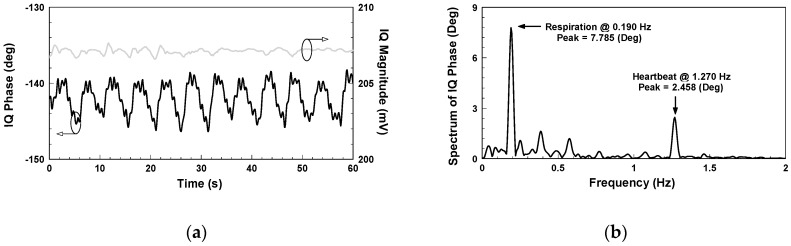
Vital sign detection results using the SIL radar: (**a**) IQ magnitude and phase; (**b**) Spectrum of IQ phase in Figure 6a.

Figure 6 shows the IQ signals measured with the architecture in Figure 2. The additional DC offsets of the two signals are 65.345 mV and −36.113 mV, respectively. Although it has been proven that clutter signals have limited influence on the SIL radar [26], hardware imperfections still cause additional DC offset. Compared with the conventional CW radar, however, the extent is significantly less. As shown in Figure 7a, the demodulated amplitude is around 207 mV. Figure 7b shows the IQ phase frequency spectrum, in which the peak values of respiration and heartbeat are 7.785 and 2.458 degrees, respectively, and the respiratory and heart rates are 11.4 and 76.2 beats per minute, respectively.

By comparison, both radars achieve similar IQ magnitude and phase results, but the SIL radar obviously consumes much less power because no LNAs are involved. The above experiment proves that the SIL radar offers better sensitivity and can reduce the number of active components to increase endurance. In addition, the SIL radar can be separated into two parts—SILO and frequency discriminator—for the detection of vital signs with a bistatic architecture [28], which further reduces the size of the wearable device and therefore provides improved comfort.

## 3. Wrist-Worn Pulse Rate Monitor Based on a Bistatic SIL Radar Architecture

**Figure 8:**
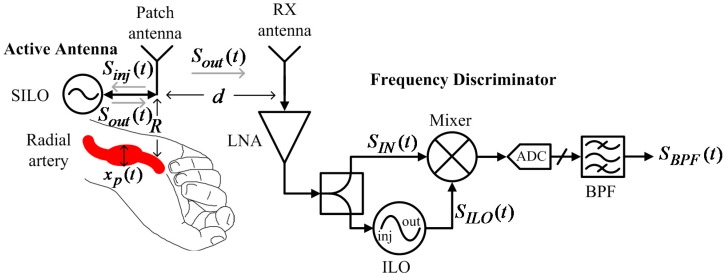
Block diagram of the proposed bistatic SIL radar architecture for a wrist pulse rate monitor.

Figure 8 shows a pulse rate monitoring system proposed by this study. The system has two parts. The first is an active antenna on a wristband that consists of a patch antenna and a SILO. The SILO’s output signal, $S_{out}\left(t\right)$, is transmitted by the patch antenna, and the echo signal from the wrist is received by the same antenna as $S_{inj}\left(t\right)$, which makes the SILO operate in a SIL state. It is worth noting that no buffer stage or other high-isolation components are required between the SILO and the patch antenna to distinguish between $S_{out}\left(t\right)$ and $S_{inj}\left(t\right)$. The Doppler effect of pulse motion causes $S_{out}\left(t\right)$ to be an FM signal carrying pulse motion information. The second part is a wireless delay discriminator, which consists of one RX antenna, one LNA, one power splitter, one mixer, and one ILO. The output signal from the SILO is received by the RX antenna, amplified, and divided to be fed to the input of the mixer and the ILO. Here, the ILO serves as the variable delay element with high frequency selectivity, and its output signal is used to pump the mixer. After the output of the mixer is sampled by the ADC and passed through the digital bandpass filter, $S_{BPF}\left(t\right)$ can then be used to determine pulse rate information.

Before commencing theoretical derivation, we first define the inherent oscillation signal, injection signal, and output signal of the SILO as $S_{osc}\left(t\right)$, $S_{inj}\left(t\right)$, and $S_{out}\left(t\right)$. When not affected by injection signals, $S_{osc}\left(t\right)$ is equal to $S_{out}\left(t\right)$. The SILO has a constant inherent frequency $\omega_{osc}$ and an amplitude $E_{osc}$. When the output signal is reflected from the wrist and received to be used as injection signal $S_{inj}\left(t\right)$ with a constant amplitude, the injection frequency $E_{inj}$. is modulated by Doppler phase shift, represented as $\omega_{inj}\left(t\right)$. Now, the SILO’s output signal, $E_{out}$, remains the same, while the frequency is influenced by the SIL effect. Based on the previous study [26], the instantaneous output frequency of the SILO can be expressed as

(4) $$\omega_{out}\left(t\right)=\omega_{osc}-\omega_{LR,osc}\text{sin}\alpha \left(t\right).$$

$\omega_{LR,osc}$ in Equation (4) is the locking range of the SILO, which can be represented as

(5) $$\omega_{LR,osc}=\frac{\omega_{osc}}{2Q}\;\times \;\frac{E_{inj}}{E_{osc}}$$

where Q is the quality factor of the SILO’s resonant circuit. In Equation (4), α(t) is the propagation phase delay caused by the SIL path, which means

(6) $$\alpha \left(t\right)=\frac{2\omega_{osc}}{c}\left(R+x_{p}\left(t\right)\right)$$

where R and $x_{p}\left(t\right)$ are the initial distance between the active antenna and the wrist and the skin surface displacement caused by pulse motion, respectively; c denotes the speed of light.

In addition to being used to illuminate the location of the radial artery and to detect the slight displacement of the skin surface, the SILO’s output signal, $S_{out}\left(t\right)$, can be wirelessly transferred to the frequency discriminator, at a distance of d from the patch antenna, to be demodulated. As shown in Figure 8, $S_{out}\left(t\right)$ is received by the RX antenna, LNA-amplified, divided into two outputs by the power splitter, and fed to the mixer and ILO. The input signal $S_{IN}\left(t\right)$ can be found as

(7) $$S_{IN}\left(t\right)=E_{IN}\text{cos}\left(\omega_{osc}\left(t-\tau_{p}\right)-\omega_{LR,osc}\int_{0}^{t-\tau_{p}}\text{sin}\alpha \left(t^{\prime }\right)dt^{\prime }+\theta_{0}\right)$$

In Equation (7), $E_{IN}$ and $\theta_{0}$ are the constant input amplitude and initial phase, respectively; $\tau_{p}$ is the transmission delay from the active antenna to the frequency discriminator, namely d/c. When ILO is injection-locked by $S_{IN}\left(t\right)$, the output signal of ILO, based on the derivation above [27], is given as

(8) $$S_{ILO}\left(t\right)=E_{ILO}\text{cos}\left(\omega_{osc}\left(t-\tau_{p}-\tau_{IL}\right)-\omega_{LR,osc}\int_{0}^{t-\tau_{p}-\tau_{IL}}\text{sin}\alpha \left(t^{\prime }\right)dt^{\prime }+\theta_{0}\right)$$

where $E_{ILO}$ is the constant output amplitude of $S_{ILO}\left(t\right)$. By comparing Equations (7) and (8), it is clear that the phase component of $S_{ILO}\left(t\right)$ is synchronized by $S_{IN}\left(t\right)$, yet injection locking provides an additional time delay, $\tau_{IL}$, which is estimated as

(9) $$\tau_{IL}=\frac{1}{\sqrt{{\omega_{LR,ILO}}^{2}-∆{\omega_{osc}}^{2}}}$$

where $\omega_{LR,ILO}$ is the locking range of the ILO; $∆\omega_{osc}$ is the frequency difference between the SILO and the ILO signal. The output signal of the mixer passes through the ADC and digital bandpass filter to remove DC components and high-order intermodulation components. According to Equation (9), by adjusting the inherent frequency of the SILO and the ILO, the following conditions can be fulfilled.

(10) $$\{\begin{array}{c} \omega_{osc}\tau_{IL}=\left(n+0.5\right)\times \pi \\ \omega_{osc}\frac{2d}{c}=n\times \pi \end{array}$$

In Equations (10), n is a natural number. Assume that $2\omega_{osc}x_{p}\left(t\right)/c\ll 1$, and the bandpass-filtered output signal can be approximated as

(11) $$S_{BPF}\left(t\right)=\frac{2G_{c}E_{IN}\omega_{osc}\omega_{LR,osc}\tau_{IL}}{c}\times x_{p}\left(t-\tau_{p}\right)$$

where $G_{c}$ is the conversion gain of the mixer. Because $\tau_{p}$ is far shorter than the cycle of physiological signals, $x_{p}\left(t-\tau_{p}\right)$ can be considered as $x_{p}\left(t\right)$. We can therefore obtain the pulse rate information of the subject from Equation (11).

## 4. Experimental Results and Discussion

**Figure 9:**
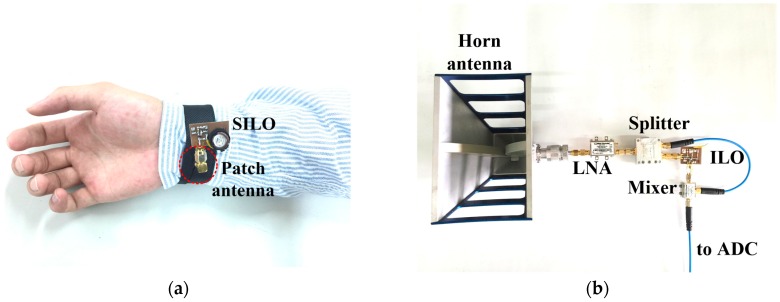
Photograph of the wrist pulse rate monitor. (**a**) Active antenna wristband; (**b**) Frequency discriminator.

Figure 9 presents the photograph of the implemented pulse rate monitoring system. According to the authors’ previous research [25], when the operating frequency doubles, the signal-to-noise ratio (SNR) gain of the SIL radar quadruples. Because the displacement fluctuations caused by the radial artery pulse are weaker than those caused by heartbeats, the prototype in this study operated at the 5.2 GHz unlicensed band to improve the detection sensitivity. As shown in Figure 9a, the SILO is based on a Colpitts oscillator and powered by a 3 V button battery. The current consumption is 9 mA, the operating frequency is 5.2 GHz, and the output power is 7 dBm. The size of the PCB is 2.5 × 2 cm^2^. The output port of the SILO is connected to a patch antenna with a gain of 7 dBi. The antenna is attached to the inner side of the wristband, indicated by the red circle of dotted lines in the figure, to detect the pulse motion. The size of the antenna is 2.2 × 2.2 cm^2^. The RF signal from the active antenna is received by the remote frequency discriminator, at a distance up to 60 cm. Figure 9b is a photograph of the frequency discriminator. The ILO design is similar to that shown in Figure 3, with the resonance circuit adjusted to operate at 5.2 GHz with an output power of 7 dBm. It can pump the mixer while providing a time delay to demodulate the FM signal received by the horn antenna. Other components are commercially available products whose key specifications are listed in Table 2.

**Figure 10:**
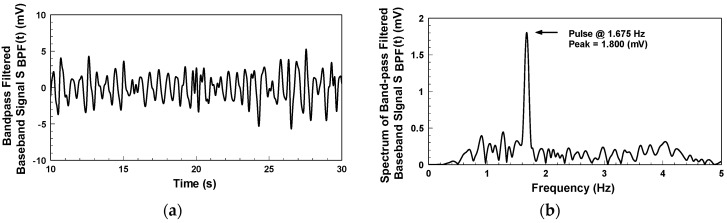
Pulse detection results for the subject wearing a short-sleeved shirt: (**a**) Bandpass-filtered baseband signal; (**b**) Spectrum of Figure 10a.

**Figure 11:**
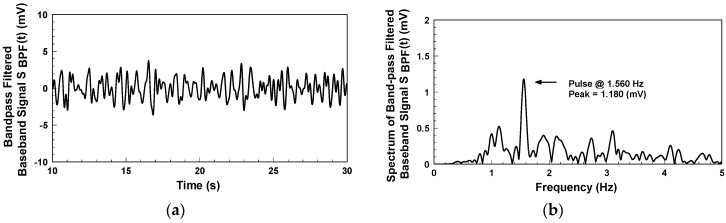
Pulse detection results for the subject wearing a long-sleeved shirt: (**a**) Bandpass-filtered baseband signal; (**b**) Spectrum of Figure 11a.

After sampling the mixer’s output signal with NI 9215 DAQ, Matlab is used for subsequent signal processing and analysis. Figure 10a indicates the detection of the bandpass-filtered output signal when the subject, who wore a short-sleeve shirt, placed his arm flat on the table. The waveform represents the skin surface displacement caused by the pulse on the subject’s wrist. The passband of the digital bandpass filter is from 0.5 to 3 Hz. Figure 10b shows the frequency spectrum of Figure 10a. The pulse rate of the subject is 100.5 beats per minute, with a peak value of 1.8 mV. This system uses electromagnetic waves as the detection medium, which can penetrate non-metal materials for pulse detection. Figure 11 shows the pulse detection results when wearing the active antenna wristband on a clothed target area clothed. As a detection result, the pulse rate is 93.6 beats per minute, with a peak value of 1.18 mV. Though this peak value is lower than that in Figure 10b, the pulse rate information is still accurate.

The relative motion between the active antenna and the wrist, such as hand-waving, fidgeting, and random body motion, would cause large fluctuations in the baseband signals and significantly lower the detection accuracy. However, compared to the vital signs, these interference signals are temporary and aperiodic, thus the joint time-frequency analysis can be utilized to establish a reliable spectrogram for long-term monitoring of the target signal with a wide range of physical activities [28]. Notably, since the modulation bandwidth of the active antenna worn on a moving subject’s wrist is generally less than 7 MHz, this remote frequency discriminator with gain-enhanced LNA could support up to 14 active antennas to concurrently detect vital signs of 14 users by simply switching the carrier frequency of the ILO. Moreover, the built-in battery can provide up to four days of antenna active time, and the battery will even last longer if utilizing a pulse-width modulation (PWM) power-supply controller, a future development possibility worth looking forward to.

## 5. Conclusions

This study uses bistatic SIL radar technology to monitor the pulse rate of a subject who wears an active antenna wristband. The radial artery pulse on the wrist causes the active antenna to output FM signals. After being demodulated by an ILO-based frequency discriminator at a remote location, the pulse rate information of the subject is obtained accurately. Based on the technology described in this study, the proposed bistatic SIL radar architecture can effectively reduce the size and power consumption of wearable devices for vital sign detection. Future research will focus on using flexible electronics to produce more comfortable wearable radars suitable for long-term monitoring for vital signs and exercise tracking.

## Figures and Tables

**Table 1:** Comparison of vital-sign sensing techniques.

Technology	Sensitivity	Sensing Style	System Complexity	Power Consumption
Accelerometer	Low	Wearable	Low	Low
Piezoelectric device	Low	Contact	Low	Low
Electrocardiography (ECG)	High	Contact	High	Low
Photoplethysmography (PPG)	High	Wearable	High	High
Pulse radar	High	Wearable	High	High
CW radar	High	Non-contact	High	Medium
SIL bistatic radar	High	Wearable	Low	Low

**Table 2:** List of components used in the wrist-worn pulse rate monitor.

Component	Model Number	Operating Frequency	Specifications
**Active antenna**			
SILO	Self-made PCB	5.1–5.3 GHz	Output power: 7 dBm
Patch antenna	Self-made PCB	5.1–5.3 GHz	Antenna gain: 7 dBi
**Frequency discriminator**			
Horn antenna	HA-08M18G-NF	0.8–18 GHz	Antenna gain: 12 dBi @ 5.2 GHz
LNA	ZX60-542LN-S+	4.4–5.4 GHz	Gain: 24 dB; Noise figure: 1.9 dB
Splitter	0120A0220800	2–8 GHz	Insertion loss: 3.6 dB
ILO	Self-made PCB	5.1–5.3 GHz	Output power: 7 dBm
Mixer	ZMX-7GR	3.7–7 GHz	Conversion loss: 5 dB; LO power: 7 dBm
